# Cost-Effectiveness of Community-Based Interventions for Hypertension Prevention and Management: A Protocol for Systematic Review, Meta-Analysis, and Quality Assessment.

**DOI:** 10.31729/jnma.v63i292.9255

**Published:** 2025-12-31

**Authors:** Reshu Agrawal Sagtani, Amrit Banstola, Shirley Crankson, Sayem Ahmed, Swornim Bajracharya, Swornim Archana Shrestha, Ghanshyam Gautam, Subhash Pokhrel

**Affiliations:** 1 Health Economics Research Group, Department of Health Sciences, Brunei University of London, UK; 2 NIHR, Global Health Research Center for Multiple long term conditions, Kathmandu Medical College, Sinamangal, Nepal; 3 School of Public Health, Patan Academy of Healtth Sciences, Lalitpur, Nepal; 4 Department of Public Health, Kathmandu University School of Medical Sciences, Dhulikhel, Nepal; 5 HERD International, Kathmandu, Nepal

**Keywords:** *cost-effectiveness*, *community-based*, *economic evaluation*, *hypertension*

## Abstract

**Introduction:**

Hypertension presents a global health challenge, contributing significantly to household and health system costs. While clinical effectiveness of hypertension interventions is well documented, evidence on the cost-effectiveness of community-based interventions remains limited. This review aims to evaluate the economic evidence of community-based interventions for hypertension prevention and management and compare cost-effectiveness estimates across diverse geographical and implementation settings.

**Methods:**

A systematic search will be conducted across databases such as Scopus, Web of Science, Cumulative Index to Nursing and Allied Health Literature Plus, American Psychological Association PsyclNFO, National Health Service Economic Evaluation Database, and Cochrane Central, covering literature up to January 2025. Grey literature and preprints will also be included. Eligible Judies will be full economic evaluations comparing two or more community-based interventions, in any language. Two reviewers will independently screen Judies using RAYYAN software. Quality assessment will be performed using validated checkMs. A meta-analysis will be undertaken contingent upon the presence of adequate homogeneity in outcomes and methodologies.

**Discussion:**

This review will highlight cost-effectiveness estimates and identify methodological and subject-specific gaps in the literature which can provide comprehensive insights to inform policy decision on CBIs for HTN. While focusing on adult populations may introduce publication bias, this will be considered during interpretation.

**Systematic review registration:**

This review is registered under PROSPERO, Indentifier: CRD420246129.

## INTRODUCTION

Hypertension is a leading global risk factor for death and disability, affecting nearly 1.4 billion adults aged 30-79 years in 2024.‘Beyond its cardiovascular complications^2^, it imposes a substantial economic burden.^3^ Direct and indirect healthcare costs can be catastrophic for households^4^ and detrimental to national economie^5^, especially in Low and Middle Income Countries (LMICs) where costs may exceed per capita health expenditure.^6^ Community-based interventions (CBIs) offer accessible, cost-effective, and culturally sensitive care^7^, leveraging local resources to improve adherence and reduce Cardiovascular disease (CVD) risks^8^, potentially averting future healthcare costs.^9^ While CBIs have shown effectiveness globally^10-12^, systematic evidence on their cost-effectiveness remains limited. Prior reviews focused on broader CVD prevention^13-15^ or high-income contexts,^16-18^ often excluding key databases and modeling studies.^19-20^ This review aims to synthesize global evidence on cost-effectiveness of CBIs for hypertension, identify reported outcomes and costs, assess economic methods and study quality, and highlight gaps to inform future research.

## METHODS

The protocol of this systematic review is registered with the International Prospective Register of Systematic (PROSPERO)^21^ registration number CRD42024612912 and its reporting is in accordance with the Preferred Reporting Items for Systematic Reviews and Meta-analyses Protocols (PRISMA-P) checklists (Supplementary file 1).^22^ If the included studies exhibit sufficient homogeneity in interventions, economic outcomes, and study characteristics , a meta-analysis will be conducted. In cases where substantial heterogeneity is identified, we will use the Synthesis Without Meta-analysis (SwiM) checklist^23^ (Supplementary file) to guide the narrative synthesis. Sources of heterogeneity will be explored, and findings will be summarized.

### ELIGIBILITY CRITERIA

Studies will be selected according to criteria around the Population, Intervention, Comparator, Outcomes(s) of the interest, and Study Design (PICOS) framework.^24^ The selection criteria has been summarized in table 1 and studies from inception (i.e., no lower date limit) to January 2025 will be considered. Studies will not be excluded on the basis of language and we will use AI tools for initial translation of non-English abstracts followed by human verification by bilingual reviewers to ensure accuracy and contextual relevance of the translated content.

**Table 1:** Selection criteria of the review based on PICOS framework.

Study characteristics	Inclusion Criteria	Exclusion Criteria
Population	All adults (aged 18 years and above). No restriction on geographical settings.	Minors (below 18 years)
Intervention	CBIs targeted at primary and secondary prevention of hypertension through primary care approach. The interventions are multicomponent and focuses on community level participation and involvement.	Interventions for management of complications of hypertension at an individual level/hospitalbased setting. Interventions for management of management of conditions co-morbid with hypertension.
Comparator	Studies which use Existing/Usual/Standard of care and also No intervention for hypertension are acceptable.	----
Outcomes	Primary: Incremental Costs Effectiveness Ratio (ICER)/Net Monetary Benefit (NMB)/Net Health Benefit (NHB)/cost per unit benefit. Secondary: types of costs, types of outcomes and methodological approaches.	Cost of illness/cost consequence/budget impact analysis
Study design/type	Intervention studies (randomized, non-randomised, quasi experimental designs); observational studies, full economic evaluation studies, modeling studies. All study designs should compare two or more interventions in terms of both costs and consequences.	Non-human research, abstracts, conference proceedings, reviews (systematic/scoping/narrative or meta-analysis), study protocols, qualitative research, case studies, partial economic evaluation, cost of illness studies.

### INFORMATION SOURCES

A structured search will be conducted across major medical, allied sciences, health economics, and grey literature databases. The included Scopus (that includes PubMed/MEDLINE, 1788 onwards), Web of Science (Core Collection, 1970 onwards), Cumulative Index to Nursing and Allied Health Literature (CINAHL) Plus (EBSCOhost, 1937 onwards), APA PsycINFO (EBSCOhost, 1872 onwards), NHS Economic Evaluations database (EED) including HTA database (Centre for Reviews and Dissemination, until March 2015), Cochrane Central Register of Controlled Trials (CENTRAL), Cost-Effectiveness Analysis (CEA) Registry (Tufts Medical Centre, 1976 onwards), Research Papers in Economics (RePEc) via EconPapers, Google Scholar (first 150 results) and GreyNet International for grey literature and Open Science Framework (OSF) for preprints.

### SEARCH STRATEGY

The search strategy will be developed in collaboration with the health sciences librarian at Brunel university of London. It will use medical subject headings (MeSH) terms and pilot searches targeting five key concepts: cost-effectiveness, community-based, prevention, management, and hypertension. A sample SCOPUS strategy is provided in (Supplementary File). Final strategies will be reported in the full review.

### SCREENING PROCEDURE

Identified records will be managed using RAYYAN software which will enable de-duplication. At the first stage, titles and abstracts will be screened by one reviewer, followed by screening of eligible articles two independent reviewers. Disagreements between the reviewers will be resolved through discussion, and if consensus cannot be reached, a third independent reviewer will be consulted to make the final decision. Full texts will be retrieved for selected articles. A PRISMA flowchart will document the screening process and reasons for exclusion.

### DATA EXTRACTION

Data extraction and risk of bias assessment will be conducted by at least two independent reviewers. Disagreements will be resolved by consensus or adjudication by a third independent reviewer. A structured extraction form will capture study details (author, year of publication, title, design, objectives, patient characteristics, sample size, location, and settings), disease characteristics (definition and measurement of hypertension), intervention characteristics (inclusion of community sectors, types of intervention, mode/level of prevention), details of comparator and study outcomes such as Incremental Cost Effectiveness Ratios (ICERs), Net Monetary Benefit (NMB), Disability Adjusted Life Years (DALYs) averted, Quality Adjusted Life Years (QALYs) gained, improved Quality of Life (QoL) outcomes and reduction in blood pressure. Additional data items pertaining to economic evaluation methods (type of evaluation, perspective, time horizon, discounting, analytical approach (trial-based/model-based of both), resource use, unit costs, types of costs, currency) and characteristics of economic model (model type, major assumptions of the model, scenario,sub-group analysis and uncertainty analysis) will be included. The data extraction form will be piloted on a sample of included studies prior to full data extraction. Any necessary adjustments will be made baseed on the feedback from the review team during this pilot phase.

### REPORTING QUALITY IN INDIVIDUAL STUDIES

Risk of bias will be assessed using the ECOBIAS^25^ checklist which covers both model-based and trial based economic evaluation studies. Methodological quality of the trial based studies will be assessed through Consensus Health Economic Criteria (CHEC)^26^ checklist, and quality and transparency of the model based economic evaluations will be assessed through Philips checklist.^27^ Reporting quality of the studies will be validated through Consolidated Health Economic Evaluation Reporting Standards (CHEERS) checklist.^28^ Quality of grey literature including government and organizational reports will be appraised using a modified version of the Authority, Accuracy, Coverage, Objectivity, Date, Significance (AACODS) checklist.^29^ To prevent discordant assessments resulting from use of multiple tools, we plan to apply each tool according to intended scope and present results from each tool separately in the review. In case of discrepancies, we will discuss their implications for the overall quality and reliability of the included studies. Quality assessment will inform interpretation but not determine exclusion. Bias assessment will be applied at the study level rather than the outcome level. Inter-rater agreement will be assessed during the quality appraisal process using percent agreement to evaluate consistency between reviewers. This will be calculated as the proportion of items on which reviewers agree, out of the total number of items assessed. Agreement above 80% will be considered acceptable. Any disagreements will be resolved through discussion or consultation with a third reviewer.

### DATA SYNTHESIS

Narrative synthesis will follow the PICO framework. Findings will be tabulated by study details, interventions, outcomes measures and conclusions. Reported ICERs and NMBs will be used as the standardized metrics for cost-effectiveness. A contextual summary table will be created referencing GRADE Economic Evidence Profile, National Institute for Health and Care Excellence (NICE), UK guidance,^30^ including study limitations, price year, incremental analyses, unicertainty and additional information. All costs will be converted to 2025 UK Pounds using the Campbell and Cochrane Economics Methods Group (CCEMG)—Evidence for Policy and Practice Information and Coordinating Centre Cost Converter (V.1.6.41).^31^ The gross domestic product destator index and purchasing power parity conversion rates will be applied to facilitate cost and incremental cost-effectiveness comparisons across studies. A random effects meta-analysis will be considered is at least three studies report comparable economic outcomes (ICERs or NMB) and share sufficient methodological similarity in terms of intervention, comparator, perspective and time horizon. Additionally, studies must provide adequate data to allow for harmonization, including currency conversion and variance estimation. Where these conditions are met, we will proceed with meta-analysis and report pooled estimates.

Forest plots will be used to visualize the extent of heterogeneity while statistical heterogeneity will be established through I^2^ statistics. We will also report Tau^2^ and Cochran Q test with P value of < 0.05 considered statistically significant (heterogeneity).

### ADDITIONAL ANALYSES

Subgroup analyses will be conducted where studies have reported cost-effectiveness by subsets of the target populations based on characteristics of study population (age, sex, education, income level), intervention type (based on level of prevention), and geographical and healthcare settings (e.g., low-income, middle income, high income countries).

### DATA MANAGEMENT

All data extraction forms, screening decisions, and relevant documentation will be securely stored in a shared, access-controlled repository-specifically, the OneDrive system of Brunel University of London. Additionally, metadata and documentation will be maintained to facilitate future audits or replication.

## DISCUSSION

Although there is evidence on the cost-effectiveness of CBIs for cardiovascular diseases, comprehensive economic evaluations specifically targeting hypertension remain limited. This review will offer valuable insights into current methodologies used in economic evaluations, the quality of available evidence, and the gaps that persist in assessing CBIs for hypertension in community settings. A deeper understanding of the factors’ instuencing cost-effectiveness is crucial for policymakers and researchers involved in designing interventions and making resource allocation decisions. Additionally, summarizing the types of costs, effectiveness measures, and economic evaluation methods will help inform future evaluations and guide decisions in both resource-rich and resource-limited settings.

Most economic evaluations adopt a specific adopt a specific perspective due to scope, resource constraints, and data availability, which may not capture all costs and benefits of an intervention. Although subgroup analyses will be conducted, variations in research methods, settings, and outcomes may limit the feasibility of a quantitative meta-analysis. Restricting the study population to adults ensures population homogeneity but excludes CBIs targeting hypertension prevention among minors a potential limitation that should be considered when interpreting findings. Any deviations from the protocol will be documented, including the date, description, rationale, and implications of the changes.

## Data Availability

The data are available from the corresponding author upon reasonable request.
